# Modulation of Cellular Biochemistry, Epigenetics and Metabolomics by Ketone Bodies. Implications of the Ketogenic Diet in the Physiology of the Organism and Pathological States

**DOI:** 10.3390/nu12030788

**Published:** 2020-03-17

**Authors:** Arkadiusz Dąbek, Martyna Wojtala, Luciano Pirola, Aneta Balcerczyk

**Affiliations:** 1 Department of Molecular Biophysics, Faculty of Biology and Environmental Protection, University of Lodz, Pomorska 141/143, 90-236 Lodz, Poland; arkadiusz.dabek@unilodz.eu (A.D.); martyna.wojtala@biol.uni.lodz.pl (M.W.); 2 INSERM Unit 1060, CarMeN Laboratory, 165 Chemin du Grand Revoyet - BP12, F-69495 Pierre Bénite CEDEX, France; luciano.pirola@univ-lyon1.fr

**Keywords:** ketone bodies, β-hydroxybutyrate, ketogenic diet, epigenetics, inflammatory response, cancer

## Abstract

Ketone bodies (KBs), comprising β-hydroxybutyrate, acetoacetate and acetone, are a set of fuel molecules serving as an alternative energy source to glucose. KBs are mainly produced by the liver from fatty acids during periods of fasting, and prolonged or intense physical activity. In diabetes, mainly type-1, ketoacidosis is the pathological response to glucose malabsorption. Endogenous production of ketone bodies is promoted by consumption of a ketogenic diet (KD), a diet virtually devoid of carbohydrates. Despite its recently widespread use, the systemic impact of KD is only partially understood, and ranges from physiologically beneficial outcomes in particular circumstances to potentially harmful effects. Here, we firstly review ketone body metabolism and molecular signaling, to then link the understanding of ketone bodies’ biochemistry to controversies regarding their putative or proven medical benefits. We overview the physiological consequences of ketone bodies’ consumption, focusing on (i) KB-induced histone post-translational modifications, particularly β-hydroxybutyrylation and acetylation, which appears to be the core epigenetic mechanisms of activity of β-hydroxybutyrate to modulate inflammation; (ii) inflammatory responses to a KD; (iii) proven benefits of the KD in the context of neuronal disease and cancer; and (iv) consequences of the KD’s application on cardiovascular health and on physical performance.

## 1. Introduction

The presence of ketone bodies (KBs) in all forms of living organisms, including *Eukaryotes*, *Prokaryotes* and *Archaea*, is a consequence of lipid metabolism; in particular, β-oxidation [1]. These low molecular weight intermediates, i.e., acetoacetate (AcAc), β-hydroxybutyrate (BHB) and acetone (Ac), act as an alternative to glucose as energy fuel [1]. Under physiological conditions, the plasma concentration of KBs in humans oscillates around 0.05–0.1 mM, whereas in the conditions of enhanced KB-production caused by prolonged exercise, starvation, carbohydrate restriction/ketogenic diet or insulin deficiency, their level can reach 5–7 mM, and in particular circumstances even 20 mM, a concentration indicative of diabetic ketoacidosis [2]. Although ketoacidosis is a pathological state, nutritional induction of mild ketonemia, due to consumption of a ketogenic diet, intermittent fasting or caloric restriction, proved beneficial in animal models, leading to improved metabolic profiles, extended lifespans and improved neurological responses. In humans, a KD may contribute to alleviating neurological disorders [3]. On the other hand, KD-induced persistent mild ketonemia rises low density lipoprotein cholesterol levels, potentially increasing the risk of cardiovascular disease [4], although the KD-induced rise in low density lipoprotein cholesterol levels is not unequivocally observed in all studies [5,6]. Here, we will discuss the effects of ketone bodies on cellular metabolism, and their link to pathophysiology, while also considering the impact of KB as epigenetic modulators, as there is a large and growing body of evidence demonstrating a role of KB, particularly β-hydroxybutyrate, in the regulation of chromatin histone post-translational modifications (PTMs), and thus in the transcriptional machinery.

BHB, also designated as D-3-hydroxybutyric acid, is the most abundant ketone body, constituting around 70% of the circulating KB pool. Quantitatively, BHB is mostly produced by the liver using acetyl-CoA derived from beta-oxidation of lipids. Acetoacetate, a BHB biosynthetic precursor, and its decarboxylation product acetone, are the two quantitatively less abundant—and unstable—ketone bodies. BHB crosses the blood–brain barrier, and can substitute glucose as fuel. Besides the brain, BHB is also used as an alternative source of energy to glucose in all extra-hepatic tissues [7,8]. BHB, besides serving as an alternative energy source to glucose, also acts as a signaling molecule involved in many cellular functions, including epigenetic regulation of gene transcription. The pleiotropic potential of BHB is also related to the occurrence of a polymerized form of BHB, poly-β-hydroxybutyrate (PHB). While PHB was first described in bacteria, in which it is found in large intracellular granules acting as energy stores [9], more recent studies demonstrated the presence of PHB in mammalian cells, where it acts to regulate intracellular signaling, mitochondrial functions and calcium channel activity [10,11,12].

## 2. Anabolism and Catabolism of Ketone Bodies

**Figure 1:**
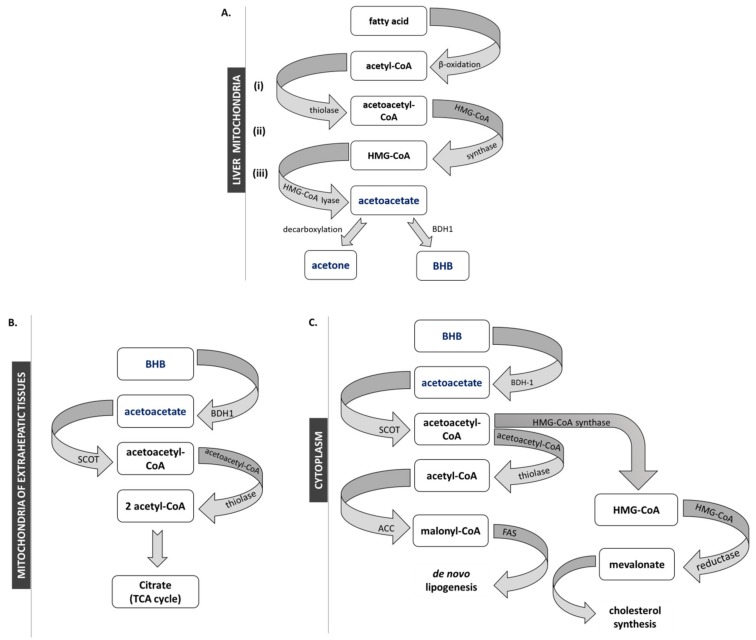
Metabolism of ketone bodies. (**A**) Synthesis of ketone bodies in the liver mitochondria. (**B**/**C**) Alternative metabolic fates of ketone bodies. (**B**) Funneling in the Krebs via succinyl-CoA:3-ketoacid coenzyme A transferase (SCOT) in mitochondria of extrahepatic tissues, and (**C**) their being used as metabolic precursors in cholesterol synthesis or de novo lipogenesis.

Ketogenesis takes place in the mitochondria of perivenous hepatocytes, and marginally in astrocytes of the brain, in Lgr5^+^ intestinal stem cells and in T-cells [8,13,14,15]. Hepatic production of ketone bodies is a physiological response to prolonged exercise, starving or reduced carbohydrate nutritional intake, but is also a pathological consequence of beta-cells failing to secrete insulin in diabetes. Under these circumstances, the liver starts producing ketone bodies from acetyl-CoA derived from the β-oxidation of fatty acids [16]. Ketogenesis is promoted when mitochondria fail to provide a sufficient amount of oxaloacetate to condense with acetyl-CoA to form citric acid and enter the Krebs cycle. Thus, acetyl-CoA is funneled through ketogenesis (Figure 1).

In the first step of ketogenesis, thiolase condensates two molecules of acetyl-CoA into acetoacetyl-CoA (AcAc-CoA), which is the substrate for β-hydroxy-β-methylglutaryl-CoA synthase 2 (HMG-CoA synthase 2), leading to the synthesis of HMG-CoA. In turn, HMG-CoA lyase metabolizes HMG-CoA to the unstable ketone body—acetoacetate (AcAc). AcAc is finally converted into stable BHB by D-β-hydroxybutyrate dehydrogenase (BDH1). Due to spontaneous decarboxylation, a fraction of the AcAc pool undergoes spontaneous decarboxylation to yield acetone, which is excreted from the body with urine and exhaled by the lungs—yielding a characteristically sweet, fruity breath. 

Transport of BHB through the plasma membrane occurs via the monocarboxylate transporter proteins (MCT). Only 3 out of 14 MCT isoforms, MCT1, 2 and 4, are involved in BHB transport. MCT expression is tissue-specific, with MCT1 being ubiquitously expressed, MCT2 being specifically expressed in the brain and kidney, and MCT4 being expressed in skeletal muscle, heart, lung and brain [17,18]. After reaching the mitochondria of the target cells, BHB is metabolized back into acetylCoA (Figure 1B). In the extrahepatic organs/tissues (i) BHB is converted to AcAc by BDH1. Then (ii) AcAc is incorporated into AcAc-CoA in a reaction catalyzed by 3-oxoacid-CoA transferase (SCOT), the *OXCT1* gene product (to avoid a futile cycle, the expression level of SCOT in hepatic cells is very low). In the final step, (iii) AcAc-CoA is transformed by acetoacetyl-CoA thiolase to two molecules of acetyl-CoA, which are consumed in the Krebs cycle or transported to the cytosol for cholesterol synthesis [1] (Figure 1C).

## 3. Ketogenesis as A Physiological Response to Starving and Prolonged Physical Exercise and as A Pathological Phenomenon in Diabetes

Production of ketone bodies is physiologically tuned to maintain physiological concentrations of BHB in the 0.05–0.1 mM range. Ketogenesis is intensified under conditions characterized by insufficient or inaccessible availability of glucose [19]. Physiologically, ketogenesis is induced by caloric restriction or prolonged exercise, resulting in accumulation and elevation of the circulating level of KBs up to 5 mM [19,20]. After ingestion of carbohydrates, the levels of ketone bodies revert to basal concentrations, as glucose is the preferable source of energy for the organism. In diabetic subjects, increased levels of ketone bodies can occur despite the high glucose plasma concentrations due to defective insulin release and impaired glucose uptake by the insulin-sensitive tissues. Under these conditions, the liver produces ketone bodies to serve the brain, heart and skeletal muscles which, due to insulin resistance and impaired glucose uptake/internalization, cannot rely on glucose supply [21]. Insulin injection may revert KB levels [19]. The in vitro studies performed on skeletal muscle isolated form mice subjected to physical exercise (swimming) for 60 min at 35 °C, have shown that 4 mM BHB significantly improves glycogen repletion in epitrochlearis muscle, the major determinant of exercise performance [22]. 

In patients with poorly controlled diabetes, increased levels of KB may lead to diabetic ketoacidosis, with KB concentrations exceeding 20 mM. Because of their acidic pH, elevated concentrations of ketone bodies observed in diabetic ketoacidosis affect the electrolyte balance, causing cell damage and dehydration, as the organism will strive to eliminate KB excess via the urine. Untreated diabetic ketoacidosis can cause coma and even death. 

## 4. Epigenetic Effects of Ketone Bodies

**Figure 2:**
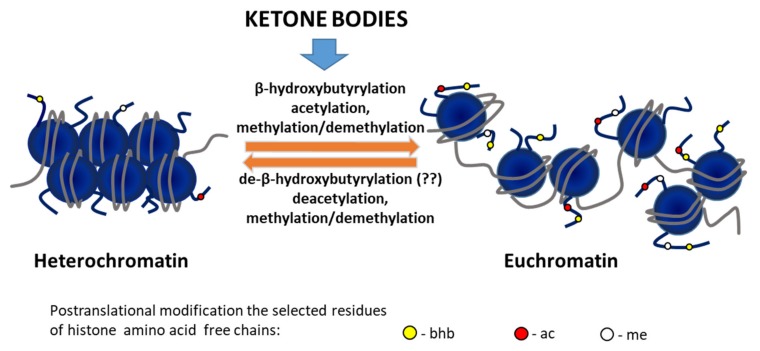
The epigenetic effect of ketone bodies on chromatin status. Pathways of modification of chromatin conformation by ketone bodies through histone posttranslational modifications (PTMs): (i) increasing the pool of acetyl-CoA as substrate for HATs, (ii) inducing changes methylation status of histones and (iii) causing β-hydroxybutyrylation per se or hyperacetylation with β-hydroxybutyrate acting as a histone deacetylase inhibitor.

Epigenetic modifications constitute a key element of regulation of gene transcription. Recent findings suggest that ketone bodies coordinate cellular functions via a novel epigenetic modification—β-hydroxybutyrylation [23,24]—that integrates the classic DNA methylation and histone covalent posttranslational modifications (PTMs), including histone lysine acetylation, methylation and histone phosphorylation and ubiquitination (Figure 2).

In response to high levels of β-hydroxybutyrate, a new type of histone posttranslational modification was identified, lysine β-hydroxybutyrylation (Kbhb), which takes place on specific lysines of histones, but also other cellular proteins, including p53 [25,26]. Using in vitro cell line models, and organs (mainly liver) from mice undergoing long-term fasting or streptozotocin-induced diabetic ketoacidosis, Xie et al. identified 44 lysines in histone proteins susceptible to β-hydroxybutyrylation, including H1K168, H2AK5/K125, H2BK20, H3K4/K9/K14/K23 and H4K8/K12 [25,27]. By genome-wide analysis (ChIP-seq) associated with transcriptional profiling, it was found that β-hydroxybutyrylation of histones produces a transcription-promoting mark enriched in active gene promoters. Moreover, the increased level of H3K9bhb, which occurs during starvation, is associated with genes upregulated in starvation-responsive metabolic pathways. These newly identified histone PTMs represent new epigenetic regulatory marks that link metabolism to gene expression, offering a new avenue to study chromatin regulation and the diverse functions of BHB in the context of important human pathophysiological states. The sequencing data revealed that H3K9bhb defines a set of upregulated genes that differ from upregulated genes bearing the H3K9ac and H3K4me3 marks, suggesting that histone Kbhb has different transcriptional-promoting functions from histone acetylation and methylation [25].

The effects of BHB on the establishment of histone posttranslational modifications other than histone Kbhb are more contradictory, especially with respect to histone acetylation. BHB was initially identified as an endogenous inhibitor of class I and IIa histone deacetylases (HDACs), which affect gene expression and chromatin modification [28]. A dose-dependent histone hyperacetylation, especially on lysines 9 and 14 of histone 3 (H3K9/K14), was identified after BHB treatment of HEK293 cells and in C57BL6/J mice maintained on caloric restriction or with elevated levels of BHB via a subcutaneous pump delivery [29,30]. However, more recent data from Chriett et al. did not confirm the function of BHB as a histone deacetylase inhibitor. Experiments performed on multiple cell lines, including HEK293 cells, myotubes (L6) and endothelial cells (HMEC-1), showed that BHB administration did not increase histone acetylation, and BHB treatment of crude nuclear extracts did not inhibit histone deacetylase catalytic activity [31]. These data are in line with the study performed by Xie et al. that presented a BHB dose-dependent induction of β-hydroxybutyrylation on multiple histone lysines with only marginal changes in the acetylation patterns [25]. 

Beyond the ongoing discussion regarding the histone deacetylase inhibitory potential of BHB, it was nonetheless demonstrated that some histone hyperacetylation following BHB treatment might be consequential to the increased intracellular acetyl-CoA pool formed by the administration of ketone bodies [32]. Besides promoting histone acetylation, such high levels of acetyl-CoA also increase the acetylation of the mitochondrial proteins [32]. Furthermore, as histone acyltransferase activity is inversely correlated to the length of the acyl chain substrate, histone acyltransferases use BHB-CoA as substrate in a less efficient manner compared to acetyl-CoA. By virtue of this, the relative abundance of lysine-hydroxybutyrylation on histone 3 (H3) and 4 (H4) is underrepresented (less than 1% of the total histone marks) compared to lysine acetylation (15–30%) [27]. 

A further complexity towards understanding of the overall effect of BHB on the chromatin acetylation patterns is added by the energetic potential of cell (i.e., the NAD^+^/NADH ratio) that is significantly modified depending on the energy fuel available to the cell: BHB or glucose. Indeed, the production of two moles of acetyl-CoA using BHB as precursor reduces only one mole of NAD^+^ to NADH, while four moles of NAD^+^ (and 4 NADH equivalents) are produced with glucose as an energy source. The excess of the NAD^+^ availability that results from a ketogenic diet likely exerts a positive influence on the redox state of the cell and potentially modulates activity of NAD^+^-dependent enzymes, including sirtuins, involved in deacetylation processes [32].

Another important aspect of epigenetic potential of the ketone bodies is their effect on the DNA and histone methylation status. Multiple studies showed that the ketogenic diet attenuates the incidence of seizures in epilepsy. However, the biological mechanism(s) whereby ketone bodies relieve the symptoms of the disease remain poorly understood, pointing at adenosine as a putatively relevant molecule curbing epilepsy progression [33]. The anticonvulsive action of a ketogenic diet was observed even after a transient administration of ketogenic therapy, and some long-term protection was apparent even after returning to a normal control diet [34]. Epigenome-wide sequencing analysis revealed significant increases in the DNA methylation levels in the hippocampi of rats suffering from chronic epilepsy [35]. In this model, a ketogenic diet therapy, beyond the attenuation of seizure progression, corrected DNA methylation-mediated changes in gene expression [35]. Detailed analysis revealed that the ketogenic diet increases adenosine’s presence, which efficiently blocks DNA methylation [23,33].

The putative contributions of BHB in the shaping of the DNA methylation profile and histone methylation status, seem to be related to the acetyl-CoA pool that, together with glycine, is needed for *S*-adenosylmethionine (SAM) synthesis. Recent studies performed on epileptic rodents showed that unbalanced dietary protein composition within a ketogenic diet may mask the anti-seizure effects of the ketogenic component of said diet, leading to an exacerbation of the seizures observed in epilepsy, possibly due to threonine deficiency, an amino acid crucial for providing a substantial fraction of intracellular glycine, and in turn, acetyl-CoA and SAM [36,37].

## 5. Signaling Pathways Linking Ketone Bodies to Protection from Oxidative Stress

Ketone bodies are not only a fat-derived energy supply form for the brain, skeletal muscle or heart under starvation or intense exercise. In 2000, Kashiwaya and co-workers found that BHB can protect neurons from oxidative damage [38]. They found that treating cells with BHB reduced the cytosolic [NADP^+^]/[NADPH] ratio and increased reduced glutathione, one of the major low molecular weight antioxidant agents in the cell. Moreover, treatment of neurons with ketone bodies revealed a decreased amount of semiquinone [38]. It was also demonstrated that in cells submitted to a pro-inflammatory stimulation by LPS treatment, BHB inhibited NF-κB by translocation and degradation of IκB-α. As NF-κB regulates expression of multiple pro-inflammatory genes, including iNOS, COX-2, TNF-α, IL-1β and IL-6, the administration of BHB to cells diminished the pro-inflammatory response to LPS [39]. 

### 5.1. Protection Against Oxidative Stress in Spinal Cord Injury

Spinal cord injury is characterized by motor, vegetative and sensitive dysfunction. The chance of recovering from such an injury is very low. A pathophysiological injury of the spinal cord causes an elevation of free radicals, which finally results in damage to surrounding tissues, causing multiple negative effects. Additionally, the blood–brain barrier, which isolates cerebrospinal fluid from blood, prevents infiltration of most antioxidants circulating in blood and does not support the recovery process. KB locally produced by astrocytes can exert a potential antioxidative effect on the spinal cord. It has been shown that ketone bodies can regulate the levels of antioxidant genes, including MnSOD and catalase, or the level of glutathione [29,40]. The ability of these genes to decrease semiquinone may also be considered as an antioxidant action, as it prevents free radical formation [33]. 

### 5.2. The Impact of Ketone Bodies/Ketogenic Diet on Alzheimer’s Disease

Alzheimer’s disease, the most significant cause of dementia, is associated with impaired glucose utilization in the brain and mitochondrial dysfunction [41]. The energy imbalance caused by the reduced glucose uptake, downregulation of glucose transporters (GLUT1) and inefficient glycolysis, alters amyloid precursor protein processing leading to the production of the neurotoxic amyloid β-peptide and consequential loss of neurons and cognitive deficits [41,42]. Ketone bodies, as an alternative energy source, are often pointed out as a possible rescue window for glucose hypometabolism in neurodegenerative disease. The studies performed on a mouse model of Alzheimer’s disease treated with the ketogenic diet showed significantly a decreased level of amyloid β-peptide in the brain and improved mitochondrial function [43,44]. A number of animal studies have shown the benefits of a ketogenic diet: better mitochondrial function, reduced oxidative stress, reduced amyloid β-peptide deposition and ameliorated tau protein pathology [43,44,45,46]. Clinical trials on human volunteers, mostly focused on mild to moderate Alzheimer’s disease patients, identified that apolipoprotein E4 (ApoE4) genotype has an effect on the outcome of ketogenic diet intake. Patients without ApoE4 allele (ApoE4(-)) presented improved short-term cognitive performance in terms of memory, language and attention, whereas ApoE4(+) patients were characterized by a reduced response to ketogenic diet treatment [47,48,49,50].

### 5.3. The Role of β-hydroxybutyrate in Ischemia/Reperfusion of Heart and Brain Injury

The two most susceptible organs to diminished oxygen concentration are the heart and brain. In both cases, insufficient delivery of oxygen and nutrients, due to arterial/coronary ischemia (in the heart) or artery blockade/leakage (in the brain), leads to severe pathological conditions, ischemic heart disease or brain stroke, respectively. The best way to minimize the induced damage is the rapid and early restoration of circulation in the damaged vessel—termed reperfusion. Paradoxically, an overly-rapid reperfusion leads to myocardial cell death, or lethal myocardial reperfusion injury [51]. Similarly, in brain tissue, reperfusion induces anaerobic glycolysis, leading to accumulation of lactate and promotion of cell death [52].

Studies performed on adult Wistar rats have shown that elevated levels of ketone bodies (AcAc and BHB), as a result of 24 h starvation, decreased ischemic and reperfusion damage in rat hearts [52,53], whereas intermittent fasting of wild type mice decreased by about 50% the infarct size caused by ischemia/reperfusion [54]. Additionally, treatment of mice with BHB caused reduction of the lipid peroxidation product malondialdehyde (MDA) in myocardium tissue [51].

Suzuki et al. have shown that in rats with induced brain ischemia, animals treated with BHB survived longer, and ATP levels in brain remained much higher than in a control group infused with saline [52]. It was also found that administration of BHB to rats with induced brain ischemia diminished the infarct area and edema formation, and decreased lipid peroxidation. Administration of BHB also mitigated neurological defects [52]. These observations prove that BHB is not only an alternative energy source but also a signaling molecule which can modulate the oxidative stress response and other metabolic pathways, leading to antioxidant protective functions in ischemic and neurological disorders. 

### 5.4. The Protective Role of β-hydroxybutyrate in Hypertension 

Hypertension, by definition a repeatedly elevated systolic blood pressure exceeding 140 over a diastolic pressure of 90 mmHg, is one of the strongest cardiovascular risk factors. Hypertension occurs very often in association with various medical conditions, including diabetes, obesity and chronic renal insufficiency, but also in association with low physical activity, cigarette smoking and an unhealthy diet [55,56]. Individuals diagnosed with hypertension are recommended to change their lifestyles into more healthy ones, both from dietary and physical exercise perspectives.

Experiments performed on rats showed that blood pressure raises with salt content in the diet and is reduced under mild ketosis [57,58]. Dietary administration of the BHB precursor 1,3-butanediol to rats on a high-salt diet reverted blood pressure to values observed in a low salt diet control group. Moreover, administration of 1,3-butanediol reduced the activity of the Nlrp3 inflammasome, a major inductor of the expression of inflammatory factors, such as caspase-1, IL-1β and IL-18 [58]. 

These results suggest that BHB can modulate the expression of the inflammasome and associated inflammatory genes via histone beta-hydroxybutyrylation, and such histone patterns and resulting gene expression levels alleviate inflammatory responses and lower blood pressure.

## 6. The link between Ketone Bodies via Nutritional Intake and Physical Performance

In healthy adults, the oxidation of ketone bodies provides only a minor fraction of total body energy, but in the heart, brain and skeletal muscles, ketone body metabolism can be significantly increased in physiological conditions such as inter-alia fasting or low carbohydrate diet [59]. In a similar manner, supplementation of medium-chain triglycerides (C8 to C10) increased plasma ketone levels (+19%) while slightly reducing glycemia (-12%) suggesting the occurrence of an alternative fuel use under mild ketosis [60]. An alternative source of ketone bodies is the ketogenic diet, where 85% of total calories come from fat, 10% form proteins and only 5%, or less, from carbohydrates.

There are social groups, especially athletes, who strive to reduce body fat, but this goal is often attained through nutritional restrictions that can have serious health consequences. Many studies have confirmed the effectiveness of the ketogenic diet in selective reduction of body fat without significant loss of non-fat body tissues [61]. However, there is disagreement in the scientific community on the perception of the influence of ketogenic diet on athletes’ endurance. It seems that the impact of ketogenic diet on aerobic performance depends on three factors: (i) exercise intensity, (ii) the status of body training and (iii) the length of the period of diet habituation. The body responds quickly to dietary changes. A study showed that only three days of high fat/moderate protein diet resulted in a decrease in physical performance of non-trained individuals [59]. Similarly, a significant decrease in endurance was observed after 6 weeks of ketogenic diet period, verified during a 45-minute cycling test [62]. Contrarily to these observations, Cox and collaborators demonstrated increased endurance performance in high-level athletes after the administration of the edible ketone body *(R)*-3-hydroxybutyl *(R)*-3-hydroxybutyrate ketone ester, a molecule of choice to achieve ketosis without using free acid BHB, or sodium BHB, which substantially affect the body’s acid and salt homeostasis respectively [63]. Athletes react differently to an increased supply of fats. A low-carbohydrate diet leads to physiological adaptation. During aerobic endurance exercise, fat becomes the dominant energy substrate, and the remaining carbohydrate resources remain intact [64]. Such observations were made both after 4 and 20 months of using the ketogenic diet. After 20-months of the ketogenic diet, a group of ultra-endurance runners showed much higher fat oxidation rates and lower oxidation of carbohydrates rates during a 180-minute run [65]. An elevated fatty acid stream leads to development of adaptive mechanisms by active tissues: increased mitochondrial β-oxidation and reduction of glucose oxidation [66,67]. The cellular mechanisms responsible for this metabolic shift are, however, not yet fully understood. It is known that increased supply of fats results in their increased availability to lipid oxidation during aerobic exercise, but we know little about the effect of a ketogenic diet on strength performance. An initial study shows that KD does not improve strength performance compared to a carbohydrate-rich diet [68].

Some studies claim that the ketogenic diet does not expose athletes to performance limitations, especially regarding strength [45]. Lambert and colleagues showed that 2-weeks of a high-fat diet (70% fat) did not reduce the strength of cyclists during an intense workout, and their strength during a moderate-intensity workout was even improved [69]. Zajac et al. provided data on the modulation of exercise metabolism by a ketogenic diet in cyclists. After 4 weeks of, K.D.; an increase in the maximum oxygen uptake and oxygen uptake at the lactate threshold level was observed. This was associated with a decrease in body weight and/or higher oxygen uptake in order to achieve the same energy efficiency as in a mixed diet. The maximum workload and the workload at lactate threshold were significantly higher after KD compared to mixed diet [70].

In conclusion, current discoveries regarding KD and aerobic exercise require further investigation into how the training status affects adaptation to KD and resulting performance [71,72].

## 7. Curbing Cancer Progression with Ketone Bodies/Ketogenic Diet

**Figure 3:**
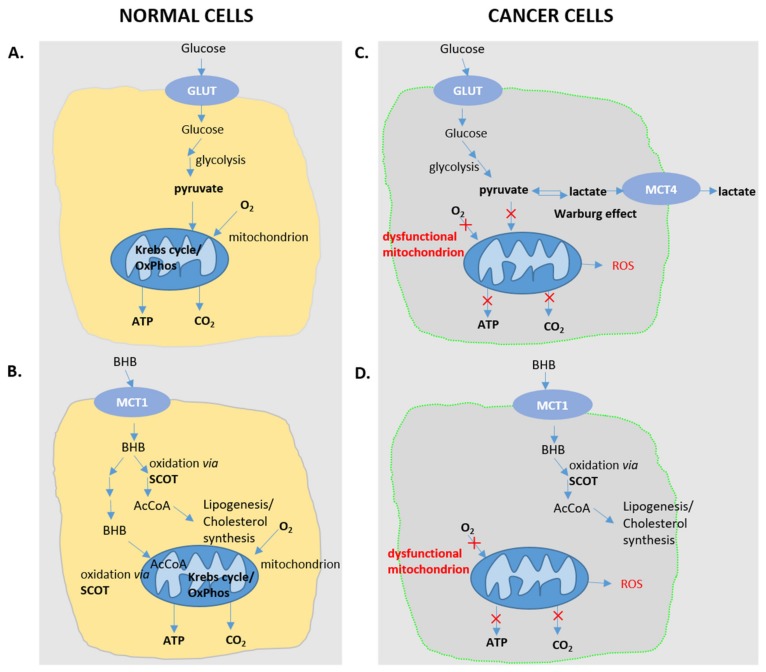
Selective use of ketone bodies by normal, non-cancerous cells can allow bypassing the glucose-induced Warburg effect in cancer cells. (**A**) In normal cells, glucose is fully oxidized through glycolysis followed by the mitochondrial Krebs cycle coupled to oxidative phosphorylation. (**C**) In cancer cells, pyruvate (the last metabolic intermediate of glycolysis) is reduced to lactate, which may serve as a precursor to sustain biosynthetic pathways. (**B**,**D**) Under conditions of glucose depletion, insulin deficiency, ketogenic diet or prolonged intensive physical activity, glucose becomes limited and cells resort to the use of ketone bodies, including BHB. (**B**) In normal cells, BHB can sustain extra mitochondrial biosynthetic pathways and serve as a AcCoA source to feed the Krebs cycle. (**D**) In cancer cells, the replacement of glucose as the primary energy source with ketone bodies (BHB) enables blunting the Warburg effect and tumor cell growth. (GLUT, glucose transporter; MCT monocarboxylate transporter; OxPhos, oxidative phosphorylation; SCOT, Succinyl-CoA: 3-ketoacid CoA transferase; ROS, reactive oxygen species; AcAc, acetoacetate).

Cancer cells need a lot of energy to support their enhanced proliferation rate. While in non-cancerous cells, carbohydrates enter glycolysis to generate pyruvate, which is then funneled into the Krebs cycle and the mitochondrial electron transport chain, tumor cells generate energy mostly by glycolysis. This phenomenon is known as the Warburg effect, which can be considered as an adaptive response allowing for carbons to be shuttled towards anabolic pathways rather than being completely oxidized in the mitochondria (Figure 3). Preference for glycolysis instead of oxidative phosphorylation to produce ATP is explained by defects of glycolytic and ketolytic enzymes in the mitochondria of tumor cells [73]. An alternative approach explaining energy production in cancer cells postulates energy transfer from normal cells in a "reverse Warburg effect" process [74]. In both cases, the tumor microenvironment is acidified, which promotes metastasis. 

In several instances, but not unequivocally, cancer progression correlates with weight loss. However, there is growing evidence that excessive body mass can also be detrimental to cancer patients [75].

Studies providing support for the anti-carcinogenic effects of the ketogenic diet (KD) implicate that mitochondrial dysfunction of cancer cells, and the concomitantly reduced expression of ketolytic enzymes, may contribute to this effect. When the blood glucose levels are falling, the cancer cells starve, whereas the normal cells change their metabolism to utilize KBs to survive. Moreover, the decrease in insulin level that accompanies the indicated ketonemic conditions correlates with a decrease in insulin-like growth factors, which promotes the proliferation of cancer cells [76]. It was shown on neuroblastoma xenografts in a CD1-nu mouse model that a ketogenic diet consisting of fat (25% medium-chain triglycerides and 75% long-chain triglycerides) and carbohydrates + protein gave the same effective therapeutic effect against neuroblastoma as did the classical ketogenic diet combined with caloric restriction [77]. 

Besides neuroblastoma, the strongest effect of the KD as an adjuvant cancer therapy has been described against glioblastoma. The currently available medical literature provides evidence for the safe application of a KD only in patients with glioblastoma [78,79]. However, some promising evidence in favor of the KD in the treatment of prostate, colon, pancreas and lung cancers has also been reported [66]. On the contrary, only limited evidence is available on the anti-cancer effect of the KD on stomach and liver cancer. Evidence of anticancer activity of KD was obtained mainly in animal models, while the support of these conclusions in humans was limited to individual cases. Additionally, controversies have arisen about the safety of using the KD. A study investigating renal cancer in a rat model with tuberous sclerosis complex indicated a carcinogenic effect of the KD long-term. Alarming results were also obtained by analyzing the effect of KD on BRAF V600E-expressing melanoma in xenograft mice. In this model, a high-fat ketogenic diet increased the level of acetoacetate in the serum, leading to increased tumor growth in human melanoma cells [80]. 

One more KD function was observed—synergistic action with chemo and radio-therapy. This is confirmed by studies on animals and a patient with glioblastoma who managed to achieve complete remission after treatment with radiotherapy, a restrictive ketogenic diet and temozolomide [81].

In conclusion, the published data suggest that ketogenic diet may be safely used as an adjuvant therapy for the selected forms of cancer, in addition to the conventional treatment. Due to the physiological differences between animals and humans, the studies on patients with various types of cancer treated with KD are needed [78]. However, it is unlikely that a ketogenic diet could be used as a primary anticancer therapy. 

## 8. Future Directions

Research aimed to decipher the impact of ketone bodies within a physiological range on multiple aspects of human physiology and pathology is a clearly expanding field which will undoubtedly further develop in the next few years, as ketone bodies—or the provisioning of endogenously synthesized ketone bodies by a ketogenic diet—hold promise to address a number of pathologies, including neurodegeneration, cancer and metabolic disease.

While the precise molecular model(s) of action of ketone bodies will require further investigation, it is now established that ketone bodies, and in particular BHB, directly impinge in transcriptional regulation via epigenetic modulation and modulate inflammatory processes. While findings in animal models are not always reproduced in the clinical setting of studies in humans, it is already proven that the ketogenic diet can be successfully used to treat pediatric epilepsy forms refractory to pharmaceutical therapy [82], as shown in several randomized controlled trials [83,84], retrospective studies [85,86] and a meta-analysis [5]. According to the current literature, it can also be speculated that ketone bodies will prove beneficial in promoting healthy aging and in alleviating the burden of metabolic disease [87], and in some instances, as a useful adjuvant during the treatment of certain cancers [78].
